# Chimeric RNA isoforms generated by diverse mechanisms from two C-type lectins modulate innate immunity in arthropods

**DOI:** 10.1073/pnas.2518148122

**Published:** 2025-10-09

**Authors:** Ying Huang, Xin Huang, Li-Hua Zhang, Qian Ren

**Affiliations:** ^a^State Key Laboratory of Climate System Prediction and Risk Management, School of Marine Sciences, Nanjing University of Information Science and Technology, Nanjing 210044, China; ^b^Jiangsu Province Engineering Research Center for Marine Bio-resources Sustainable Utilization, College of Oceanography, Hohai University, Nanjing 210024, China; ^c^Jiangsu Province Engineering Research Center for Aquatic Animals Breeding and Green Efficient Aquacultural Technology, College of Marine Science and Engineering, Nanjing Normal University, Nanjing 210023, China

**Keywords:** *Macrobrachium nipponense*, C-type lectin, chimeric RNAs, innate immunity

## Abstract

Chimeric RNA formation represents a critical mechanism for expanding protein functional diversity, yet its role in invertebrate immune adaptation remains poorly characterized. Here, we report that two C-type *lectin* genes (*MnLec2* and *MnLec3*) from distinct genomic loci in the oriental river prawn *Macrobrachium nipponense* undergo positionally flexible chimeric RNA formation via alternative trans-splicing and transcriptional slippage, generating 11 structurally diverse chimeric isoforms (*MnLec1*, *MnLec4–13*) with bidirectional exon joining. Crucially, pathogen challenges reprogram chimeric RNA frequencies to shift immune equilibrium, universally suppressing detrimental *MnLec9* while promoting protective *MnLec7* formation. Functional dissection confirms dual-action pathogen suppression, recombinant MnLec7 (rMnLec7) suppresses white spot syndrome virus replication by upregulating *antimicrobial peptides* and RNAi effectors, while accelerating *Vibrio parahaemolyticus* clearance and improving survival. Conversely, suppression of MnLec9 removes its immunosuppressive activity, synergistically enhancing host defense. This coordinated isoform rebalancing enables effective pathogen clearance. Thus, positional flexibility in chimeric RNA formation generates antagonistic isoforms that maintain immune homeostasis and deploy targeted defense upon infection, revealing an adaptive transcriptional strategy in arthropods.

Chimeric RNA formation, driven by chromosomal rearrangements (genomic events) or aberrant RNA processing (e.g., trans-splicing), generates novel transcripts by merging sequences from distinct parental genes into chimeric constructs (1, 2). This process facilitates the emergence of proteins with neofunctionalized domains pivotal to evolutionary innovation, particularly in immune adaptation (3). In arthropods, lectins function as key pattern recognition receptors that orchestrate innate immunity against pathogens (4). However, evidence for lectin diversification through chimeric RNA events remains scarce, obscuring their functional evolution in immune pathways. Here, we report that the oriental river prawn, *Macrobrachium nipponense*, exhibits positional flexibility in generating chimeric C-type *lectin* RNAs. *MnLec2* and *MnLec3* from distinct genomic loci produce diverse chimeric transcripts through transcriptional and posttranscriptional mechanisms, yielding up to 11 chimeric *lectin* isoforms. These chimeric RNAs demonstrate divergent expression levels following pathogen challenge, implicating important roles in immune regulation.

## Results

### Diverse Chimeric RNAs Arise from Positional Flexibility Between MnLec2 and MnLec3.

**Fig. 1.**
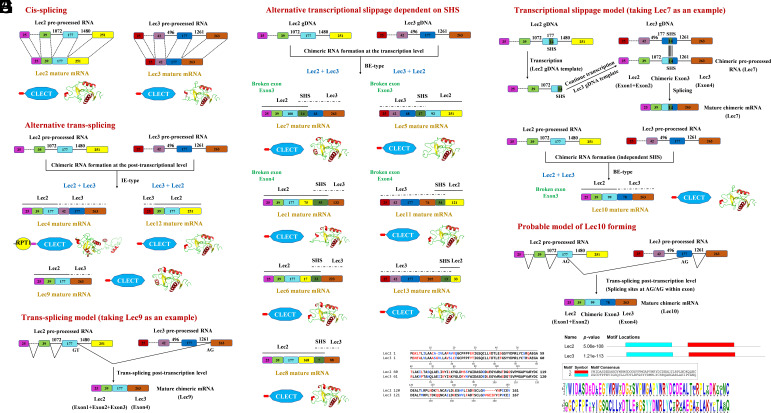
Mechanisms and structural diversity of chimeric RNAs derived from *MnLec2* and *MnLec3*. (*A*) Canonical cis-splicing generates mature *MnLec2* and *MnLec3* mRNA transcripts. (*B*) Alternative trans-splicing yields IE-type chimeric RNAs (*MnLec4*, *MnLec9*, *MnLec12*). (*C*) Posttranscriptional trans-splicing model is illustrated using *MnLec9* as representative. (*D*) Transcriptional slippage is dependent on SHS generating BE-type chimeric RNAs (*MnLec7*, *MnLec1*, *MnLec6*, *MnLec8*, *MnLec5*, *MnLec11*, *MnLec13*). (*E*) Transcriptional slippage mechanism is exemplified by *MnLec7* formation. (*F*) Noncanonical trans-splicing produces *MnLec10*. (*G*) Proposed model shows *MnLec10* formation via AG/AG splicing sites within exons. Structural annotations: exons (colored boxes; lengths in nucleotides), introns (lines), SHS (green domains), unresolved regions (dashed lines). (*H*) Sequence alignment of MnLec2 and MnLec3. Identical residues (black), conserved substitutions (blue), nonconserved residues (red).

Transcriptomic analysis of *M. nipponense*, combined with sequencing of cDNA clones derived from mRNA, revealed that two C-type *lectin* genes, *MnLec2* and *MnLec3*, underwent extensive chimeric RNA formation, generating a diverse repertoire of 11 chimeric *lectin* isoforms (*MnLec1*, *MnLec4–13*). Sequencing of multiple cDNA clones demonstrated remarkable positional heterogeneity in these chimeric transcripts. While canonical cis-splicing produced mature *MnLec2* and *MnLec3* mRNAs (Fig. 1*A*), two principal chimeric RNA generation mechanisms were identified. Intact exon (IE)-type chimeras arose from trans-splicing of intact exons at the posttranscriptional level, joining full-length exons from parental genes. *MnLec4* combined *MnLec2* exons 1–3 with *MnLec3* exons 2–4, *MnLec9* contained *MnLec2* exons 1–3 with *MnLec3* exon 4, and *MnLec12* joined *MnLec3* exon 1 with *MnLec2* exons 2–4 (Fig. 1 *B* and *C*). Broken exon (BE)-type chimeras formed via transcriptional slippage or noncanonical trans-splicing. Transcriptional slippage initially created chimeric preprocessed RNA with hybrid exons that matured after intron removal. All BE-type isoforms (except *MnLec10*) contained short homologous sequences (SHS) shared by *MnLec2* and *MnLec3*. Transcriptional slippage occurred at SHS sites during transcription, producing diverse chimeric preprocessed RNAs. *MnLec7* comprised *MnLec2* exons 1–2, a broken exon containing SHS, and *MnLec3* exon 4. *MnLec1*, *MnLec6*, and *MnLec8* shared *MnLec2* exons 1–3 but possessed distinct broken exons with different SHSs. *MnLec5* consisted of *MnLec3* exons 1–2, a broken exon, and *MnLec2* exon 4. *MnLec11* and *MnLec13* contained *MnLec3* exons 1–3 plus a broken exon (Fig. 1 *D* and *E*). Notably, *MnLec10* represented a mechanistic outlier, forming without SHS via noncanonical trans-splicing at AG/AG sites within exons (combining *MnLec2* exons 1–2, a broken exon and *MnLec3* exon 4; Fig. 1 *F* and *G*). Critically, chimeric RNAs formed bidirectionally (*MnLec2*→*MnLec3*: e.g., *MnLec4*/*7*/*9*; *MnLec3*→*MnLec2*: e.g., *MnLec5/12*). High sequence similarity between *MnLec2* and *MnLec3* facilitated SHS dependent transcriptional slippage (Fig. 1*H*). SHS mediated broken-exon formation in most BE-type isoforms (*MnLec10* excluded). Genomic DNA sequencing confirmed *MnLec2* and *MnLec3* resided at distinct loci, establishing that chimerism arose from RNA-level mechanisms, not genomic fusion.

### Pathogen Induction Patterns of MnLec2-MnLec3 Chimeric Lectins Reveal Functional Diversity.

**Fig. 2.**
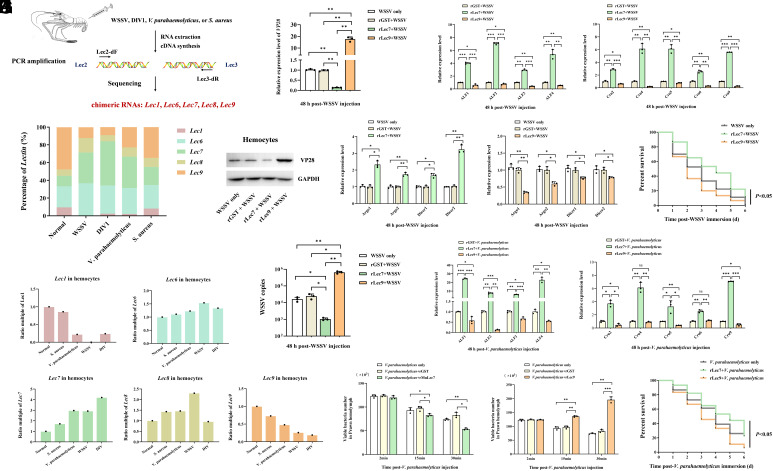
Functional divergence of MnLec2-MnLec3 chimeric lectins in antiviral and antibacterial immunity. (*A*–*C*) Differential induction of chimeric *lectins* (*MnLec1*, *MnLec6–9*) in *M. nipponense* following challenge with WSSV, DIV1, *V. parahaemolyticus*, or *S. aureus*. Relative frequencies are determined by PCR cloning/sequencing and normalized to healthy controls (dashed line = 1). The color legend shown in panel 2*B* applies to 2*C*. (*D* and *E*) Expression levels of VP28 gene (RT–qPCR) and protein (Western blot) in hemocytes at 48 h post-injection (hpi). Prawns receive WSSV preincubated with rMnLec7 or rMnLec9. GAPDH serves as a loading control. (*F* and *G*) Transcript levels of *AMP* genes (*ALF1–4*, *Crus2*, *Crus4–6*, and *Crus9*) and RNAi effectors (*Argo1–2*, *Dicer1–2*) in WSSV-challenged prawns. (*H*) WSSV genomic copies in hemocytes at 48 hpi. (*I*) Survival rates of WSSV-infected prawns are monitored over 6 d (log-rank test). (*J*) Expression of *AMPs* in prawns injected with *V. parahaemolyticus* plus rMnLec7 or rMnLec9 at 48 hpi. (*K*) In vivo bacterial clearance assay. Hemolymph bacterial loads are quantified at 2, 15, and 30 min postinjection. (*L*) Survival rates of *V. parahaemolyticus*–infected prawns (6 d). Asterisks denote statistical significance versus control (*P < 0.05, **P < 0.01, ***P < 0.001). Data represent mean ± SD (n = 3 biological replicates for *D*–*L*); *β-actin* and *GAPDH* serve as internal controls.

Pathogen challenge universally modulated chimeric RNA frequencies in *M. nipponense*. White spot syndrome virus (WSSV), Decapod iridescent virus 1 (DIV1), *Vibrio parahaemolyticus*, and *Staphylococcus aureus* consistently altered chimeric RNA frequencies, suppressing *MnLec1*/*MnLec9* and elevating *MnLec6*/*MnLec7*, with all pathogens increasing *MnLec7* and decreasing *MnLec9* (Fig. 2 *A*–*C*). Functional characterization showed recombinant MnLec7 (rMnLec7) suppressed WSSV infection by reducing VP28 transcription (Fig. 2*D*) and protein levels (Fig. 2*E*), and via upregulation of *antimicrobial peptides* (*AMPs*, *anti-lipopolysaccharide factor 1–4* (*ALF1–4*), *Crustin 2* (*Crus2*), *Crus4–6*, and *Crus9*; Fig. 2*F*) and RNAi effectors (*Argonaute1–2*, *Dicer1–2*; Fig. 2*G*), which lowered viral copies (Fig. 2*H*) and enhanced survival rates (Fig. 2*I*). Conversely, rMnLec9 exacerbated WSSV pathogenesis, elevating VP28 (Fig. 2 *D* and *E*), suppressing immune effectors (Fig. 2 *F* and *G*), increasing viral load (Fig. 2*H*), and mortality (Fig. 2*I*). Against *V. parahaemolyticus*, rMnLec7 induced *AMPs* (Fig. 2*J*), accelerated bacterial clearance (Fig. 2*K*), and improved survival rates (Fig. 2*L*), whereas rMnLec9 suppressed *AMPs* (Fig. 2*J*), impaired clearance (Fig. 2*K*), and increased mortality (Fig. 2*L*). Thus, chimeric RNA positional flexibility generated antagonistic isoforms. rMnLec7 enhanced immunity, while rMnLec9 suppressed it.

## Discussion

Chimeric RNA formation can generate chimeric RNAs at the genomic or posttranscriptional level through trans-splicing. However, our study reveals that positional flexibility in chimeric RNA formation between *MnLec2* and *MnLec3* generates up to 11 chimeric *lectins* via at least two distinct mechanisms. IE-type chimeric RNAs (e.g., *MnLec4*, *MnLec9*) arise from canonical trans-splicing of intact exons at the posttranscriptional level, producing mature chimeric RNA. In contrast, BE-type chimeric RNAs (e.g., *MnLec1*, *MnLec7*) depend on SHS from two homologous genes and first produce chimeric preprocessed RNA at the transcriptional level. Previous research observed “trans-splicing via microhomology” in *Drosophila* chimeric RNAs (5), while transcriptional slippage generates immune receptor diversity in mollusks (6). SHS acts as a molecular guide for exon joining, akin to microhomology-mediated end joining (7). Here, BE-type chimeric RNAs form through transcriptional slippage (evidenced by chimeric preprocessed RNA detection) followed by splicing. Notably, MnLec10 represents a mechanistic outlier, its broken exon 3 lacks SHS and likely forms via noncanonical trans-splicing at AG/AG sites within exons, indicating SHS-independent plasticity. While SHS are the primary drivers of BE-type chimeric RNA formation, the SHS-independent generation of MnLec10 (containing BE) via noncanonical trans-splicing reveals the existence of auxiliary mechanisms. Such events are likely stochastic and less efficient due to rare alignment of splice-compatible sites, analogous to Alu-element-driven exon shuffling in vertebrates (8). Thus, although SHS dominates chimeric RNA production, trans-splicing provides secondary routes for structural innovation. The positional independence of *MnLec2* and *MnLec3* enables combinatorial flexibility through transcriptional/posttranscriptional mechanisms, expanding immune receptor diversification beyond gene duplications. Although current events occur between homologous genes, the mechanistic basis—trans-splicing for IE-types and transcriptional slippage for BE-types—could theoretically enable chimeric RNA formation between nonhomologous genes if they share microhomology or splice-compatible sites. Nevertheless, the requirement for SHS or splice-site compatibility inherently biases diversification toward homologous families, balancing evolutionary innovation with functional feasibility.

The universal reprogramming of chimeric isoforms by diverse pathogens exemplifies the “focused efficacy” paradigm of invertebrate innate immunity. Unlike antigen-specific adaptive responses in vertebrates, this reflects pattern-specific recalibration. *MnLec7* recognizes conserved pathogen-associated molecular patterns to broadly enhance defense, while suppressing *MnLec9* removes immunosuppressive vulnerability. Despite the limited repertoire of germline-encoded receptors in innate immunity, the host can generate multiple chimeric RNA transcripts to combat foreign pathogens (9). Functional analyses confirm synergistic pathogen suppression, rMnLec7 enhances immunity by suppressing WSSV replication and accelerating bacterial clearance through upregulation of *AMPs*/RNAi effectors. Concurrently, pathogen-induced *MnLec9* downregulation ablates its proinfection activity, synergistically augmenting host defense. Thus, positional flexibility in chimeric RNA formation establishes functional antagonism, MnLec9 acts as a “homeostatic brake” under steady-state conditions, akin to *Drosophila* PGRP-LB (10), while infection triggers coordinated rebalancing (upregulation of *MnLec7* and downregulation of *MnLec9*) for targeted defense. This consolidates bidirectional immune regulation through chimeric RNA formation involving two homologous genes from distinct loci. Our study establishes chimeric RNA formation as a transcriptional strategy for expanding invertebrate immune capacity, bridging structural diversity to functional antagonism. Future studies should explore whether pathogens actively manipulate chimeric RNA frequencies to evade defense, as seen in viral hijacking of mammalian RNA splicing (11).

## Materials and Methods

Detailed experimental procedures are provided in *SI Appendix*.

## Supplementary Material

Appendix 01 (PDF)

## Data Availability

All study data are included in the article and/or *SI Appendix*.
