# Supplementary material for: Chimeric RNA isoforms generated by diverse mechanisms from two C-type lectins modulate innate immunity in arthropods
Source: Proc Natl Acad Sci U S A. 2025 Oct 9;122(41):e2518148122. doi: 10.1073/pnas.2518148122 (PMC12541431; doi:10.1073/pnas.2518148122)
Supplement: Supplementary file 1 — Appendix 01 (PDF) [file pnas.2518148122.sapp.pdf]

Supporting Information for

Chimeric RNA isoforms generated by diverse mechanisms from two C-type lectins modulate innate immunity in arthropods

Ying Huang [a,b], Xin Huang [c], Li-Hua Zhang [c], Qian Ren [a,*]

[a] *State Key Laboratory of Climate System Prediction and Risk Management, School of Marine Sciences, Nanjing University of Information Science and Technology, Nanjing, 210044, China.*

[b] *Jiangsu Province Engineering Research Center for Marine Bio-resources Sustainable Utilization, College of Oceanography, Hohai University, Nanjing, 210024, China.*

[c] *Jiangsu Province Engineering Research Center for Aquatic Animals Breeding and Green Efficient Aquacultural Technology, College of Marine Science and Engineering, Nanjing Normal University, Nanjing, 210023, China.*

*** Corresponding author:**

E-mail: renqian0402@126.com (Qian Ren)

This PDF file includes:

SI Materials and Methods

SI References

SI Materials and methods

Experimental animals and pathogens

Healthy prawns *Macrobrachium nipponense* (3–4 g) were obtained from Xianlin Aquatic Products Market (Nanjing, China). After a seven-day acclimation period in aerated tanks at 24 ± 1 °C, the prawns were used for experiments. *Vibrio parahaemolyticus* and *Staphylococcus aureus* were maintained in our laboratory culture collection. Decapod iridescent virus 1 (DIV1) and white spot syndrome virus (WSSV) were provided by Shandong University and Zhejiang University, respectively.

Immune challenge and sample collection

Prawns were randomly allocated into five groups: control (PBS-injected), WSSV-challenged, DIV1-challenged, *V. parahaemolyticus*-challenged, and *S. aureus*-challenged. The challenged groups received 100 μL injections containing WSSV (10^6 copies/mL), DIV1 (10^7 copies/mL), *V. parahaemolyticus* (3.0×10^7 CFU/mL), or *S. aureus* (3.0×10^7 CFU/mL). Control prawns received 100 μL PBS (phosphate-buffered saline; 140 mM NaCl, 2.7 mM KCl, 10 mM Na_2HPO_4, 2 mM KH_2PO_4; pH 7.4). Hemolymph was collected at 0 and 72 h post-injection (hpi) for WSSV and DIV1 challenges, and at 0 and 30 min post-injection for *V. parahaemolyticus* and *S. aureus* challenges using 1 mL syringes preloaded with 500 μL anticoagulant (1.47 g glucose, 0.48 g citric acid, 1.32 g trisodium citrate in 100 mL ddH_2O; pH 7.3). Samples were centrifuged (2,000 ×g, 10 min, 4 °C) to isolate hemocytes, which were stored in RNA stabilization solution at −80 °C.

RNA extraction, cDNA synthesis, and genomic DNA (gDNA) isolation

Total RNA was extracted from hemocytes using a High-purity Total RNA Rapid

Extraction Kit (BioTeke, China). RNA integrity was verified by 1% agarose gel electrophoresis, with concentration and purity determined spectrophotometrically (NanoDrop 2000; Thermo Fisher Scientific, USA). cDNA was synthesized from 1 μg RNA using TransScript All-in-One First-Strand cDNA Synthesis SuperMix with One-Step gDNA Removal (TransGen Biotech, China), followed by a 5-fold dilution for qPCR analysis. gDNA was isolated using the NucleoSpin Tissue Kit (Clontech, USA).

Full-length cDNA cloning and gDNA amplification

Partial *lectin* sequences (*MnLec2*, *MnLec3*) were identified from transcriptome data of *M. nipponense*. 5’- and 3’-RACE-ready cDNA was prepared using the SMARTer® RACE Kit (TaKaRa, Japan). Gene-specific primers (*MnLec2*-R: 5’-TCTCGCTGACTTCATACGTTCCCTTTT-3’, *MnLec3*-F: 5’-GCTCTGTTGCGTATCTGCAAGTAACGA-3’, *MnLec3*-R: 5’-AAGCACTTTGGATGTCTGTCCATCACG-3’) amplified terminal fragments using the Advantage 2 PCR Kit (TaKaRa, Japan) under the following program: 94 ℃ for 30 s and 72 ℃ for 3 min (5 cycles); 94 ℃ for 30 s, 70 ℃ for 30 s, and 72 ℃ for 3 min (5 cycles); and 94 ℃ for 30 s, 68 ℃ for 30 s, and 72 ℃ for 3 min (25 cycles). Genomic sequences were amplified using primers (*MnLec2*-gF1: 5’-TCACATAGGACTACTTGTTG-3’ and *MnLec2*-gR1: 5’-CAAGTATGAGTAAGCCTCAG-3’; *MnLec2*-gF2: 5’-CCGGAAGTTACTACGACATGAG-3’ and *MnLec2*-gR2: 5’-GTCGAGAGGCCAATAACAAGA-3’; *MnLec3*-gF1: 5’-CTGGTGTCCTTCTAGCTGTTTC-3’ and *MnLec3*-gR1: 5’-TGGTGCAATACAGTCTCATGTC-3’; *MnLec3*-gF2: 5’-TCGGAAGCTACTACGACATGAG-3’ and *MnLec3*-gR2: 5’-CACCGTCGAACACAAACATAAA-3’) in 25 μL reactions containing 12.5 μL 2 × Taq PCR Master Mix (Generay Biotech, China), 1 μL each primer, 1 μL gDNA, and 9.5 μL double-distilled water (ddH_2O). Thermocycling conditions were 94 ℃ for

2 min; 35 circles of 94 °C for 30 s, 53 °C for 45 s, and 72 °C for 3 min; followed by 72 °C for 10 min. Partial sequences were obtained via genome walking (Universal GenomeWalker™ 2.0 Kit; Clontech, USA) using primers (*MnLec2*-walk-R1: 5’-TGCCTGGAACAGTAGAGTCTCATGTCG-3’, *MnLec2*-walk-R2: 5’-AGGCACTG GCTGCCGATCACCTCAAAT-3’, *MnLec3*-walk-R1: 5’-GCCACTCTGGTGCAATA CAGTCTCATG-3’, *MnLec3*-walk-R2: 5’-TTGGCGAAGTCAGGCGTGTCTTTGTT G-3’). All amplicons were sequenced (Springen, China).

Chimeric MnLec sequence amplification

Primers (*MnLec2*-dF: 5’-TGCCCGTTTCCATTTGAGGTG-3’ and *MnLec3*-dR: 5’-CGCGATTTCGCAGACTGGAC-3’; *MnLec3*-dF: 5’-ATGAGTAACTTTGCCCT TGT-3’ and *MnLec2*-dR: 5’-AAGGGGAACTTCGCAGATTG-3’) were used to amplify chimeric *lectins* from hemocyte cDNA in 25 μL reactions containing 2.5 μL 10 × ExTaq Buffer, 2 μL dNTPs, 1 μL each primer (10 μM), 1 μL cDNA, 0.5 μL ExTaq Polymerase (TaKaRa, Japan), and 17 μL ddH_2O. Cycling conditions were 94 °C for 3 min; 30 cycles of 94 °C for 30 s, 56 °C for 45 s, 72 °C for 30 s; and a final extension at 72 °C for 5 min. Sequencing characterized the chimeric RNAs. The frequency of each chimeric *lectin* in approximately 50 clones from the control group or experimental groups (WSSV, DIV, *V. parahaemolyticus*, or *S. aureus* group) was calculated. To evaluate changes after pathogen challenge, the ratio of each *lectin* in the control group was set to 1, and the ratio in each experimental group was expressed relative to the control.

Bioinformatics analysis

The sequences of *MnLec1–13* have been deposited in the NCBI GenBank under

accession numbers PX104956–PX104968. Homologs were identified using BLAST (NCBI, http://www.ncbi.nlm.nih.gov/BLAST/). Amino acid sequences were translated via ExPASy Translate (https://web.expasy.org/translate/). Protein domains were predicted with SMART (http://smart.embl-heidelberg.de/). Tertiary structures were modeled with SWISS-MODEL (https://swissmodel.expasy.org/). Multiple alignments were performed with DNAMAN.

Protein expression and purification

The coding sequences of *MnLec7* and *MnLec9* were amplified using primers (MnLec7-6p-2-exF: 5'-GGATCCCCAGGAATTCCCTGCCCGTTTCCATTTGAGGTG-3' and MnLec7-6p-2-exR: 5'-GATGCGGCCGCTCGAGTTACGCGATTTCGCAGACTGGAC-3'; MnLec9-6p-2-exF: 5'-GGATCCCCAGGAATTCCCTGCCCGTTTCCATTTGAGGTG-3' and MnLec9-6p-2-exR: 5'-GATGCGGCCGCTCGAGTTACGCGATTTCGCAGACTGGAC-3'). Amplified fragments and the pGEX-6p-2 vector (Novagen, Germany) were digested with *EcoR* I and *Xho* I (NEB, USA), then ligated at 37 °C for 30 min. Recombinant plasmids were transformed into *Escherichia coli* BL21 (DE3). Positive clones were induced with 0.5 mM isopropyl β-D-1-thiogalactopyranoside (IPTG) at 37 °C with 200 rpm shaking. After 6 h induction, cells were harvested by centrifugation (6,000 ×g, 10 min, 4 °C), resuspended in PBS, and lysed by sonication. Glutathione S-transferase (GST) protein served as the control. Recombinant proteins were purified via GST Bind Resin (Novagen, Germany), analyzed by 12% SDS-PAGE, and quantified with the Bradford Protein Assay Kit (Jiancheng, China).

Recombinant protein-pathogen incubation and immune gene expression detection

Prawns were injected with mixtures of rMnLec7 or rMnLec9 (500 µL, 500 µg/mL) plus 500 µL WSSV (10^6 copies/mL) or *V. parahaemolyticus* (3×10^7 CFU/mL). At 48 hpi, hemocyte RNA was extracted for real-time quantitative PCR (RT-qPCR) using the TransStart® Top Green qPCR SuperMix (TransGen Biotech, China). Transcript levels of *VP28*, *antimicrobial peptide* genes (*MnALF1*, *MnALF2*, *MnALF3*, *MnALF4*, *MnCrus2*, *MnCrus4*, *MnCrus5*, *MnCrus6*, *MnCrus9*), and RNAi effectors (*MnArgo1*, *MnArgo2*, *MnDicer1*, *MnDicer2*) were quantified. Control groups received either the pathogen alone or the pathogen mixed with rGST protein (500 µg/mL). Primer sequences were *VP28*-qF: 5’-AGCTCCAACACCTCCTCCTTCA-3’ and *VP28*-qR: 5’-TTACTCGGTCTCAGTGCCAGA-3’; *MnALF1*-qF: 5’-GTGGTGCCCAGGATGGACTT-3’ and *MnALF1*-qR: 5’-AGAGGATGGTGGAGGAAATT-3’; *MnALF2*-qF: 5’-AGAACCACCTGAACCCAACG-3’ and *MnALF2*-qR: 5’-TGACAGATTAAGCCAGCCCC-3’; *MnALF3*-qF: 5’-GTCGATGGAGTGTATGATGAGG-3’ and *MnALF3*-qR: 5’-GTAGTGCAGCTCGAGTCTTT-3’; *MnALF4*-qF: 5’-GGCAGAGGGCCAAGAATTAG-3’ and *MnALF4*-qR: 5’-GAATTCCAAGTCACCTGTCTCC-3’; *MnCrus2*-qF: 5’-TTTGGTTTCTGGCATTTCC-3’ and *MnCrus2*-qR: 5’-CTTGTTGCTGTCACCGCTC-3’; *MnCrus4*-qF: 5’-GGAATTAGAAGGGCCCGTCGG-3’ and *MnCrus4*-qR: 5’-TCATAGCAGCACTTGTCAGCG-3’; *MnCrus5*-qF: 5’-ACACCCCAATCACCCCCCA-3’ and *MnCrus5*-qR: 5’-TGCCTTGAAACGGCTCCCT-3’; *MnCrus6*-qF: 5’-CTCCGTGTCCTCCCATACC-3’ and *MnCrus6*-qR: 5’-AGTTCCCTGTCGACTTCCT-3’; *MnCrus9*-qF: 5’-GGTGTTCTAGTTGCCGTTACA-3’ and *MnCrus9*-qR: 5’-GTGGTTCCGGGCAATTCTAA-3’; *MnArgo1*-qF: 5’-CGACGGTGGATGTTGGTATTA-3’ and *MnArgo1*-qR: 5’-GACTAGGTCGACTTGTTCCTTG-3’; *MnArgo2*-qF: 5’-GGATGGAGTTGGAAGGTCTAAG-3’ and *MnArgo2*-qR: 5’-GTACCCTGAATACCCTGATGTG-3’; *MnDicer1*-qF: 5’-GGAGACGTGTTTGAGAGTGTAG-3’ and

MnDicer1-qR: 5'-GCTCTATTTCTCGGCACATCA-3'; *MnDicer2*-qF: 5'-CCCACTA GAGCATCCTGTTTC-3' and *MnDicer2*-qR: 5'-ATCAGAGCCTCAGTGCAATC-3'. Reactions (10 μL) contained 5 μL 2 × SuperMix, 0.4 μL each primer (10 μM), 1 μL cDNA, and 3.2 μL PCR-grade water. Thermocycling parameters were 95 °C for 60 s; 40 cycles of 95 °C for 15 s and 60 °C for 30 s. Melting curve analysis (60-95 °C) confirmed amplification specificity. *β-actin* (*Mnβ-actin*-qF: 5'-TATGCACTTCCTCAT GCCATC-3' and *Mnβ-actin*-qR: 5'-AGGAGGCGGCAGTGGTCAT-3') and *glyceraldehyde-3-phosphate dehydrogenase* (*MnGAPDH*-qF: 5'-CGGTATCCAGCTC AGCAAA-3' and *MnGAPDH*-qR: 5'-CTGCATGTGCTTCAACAAGTC-3') served as endogenous controls. Data from three biological replicates were analyzed using the $2^{-\Delta\Delta CT}$ method (1), with statistical significance ($P < 0.05$) determined by Student's *t*-test.

VP28 protein detection by Western blot

Prawns injected with rMnLec7/WSSV or rMnLec9/WSSV mixtures were sampled at 48 hpi. Hemocyte proteins were extracted using the One Step Animal Tissue/Cell Active Protein Extraction Kit (Sangon Biotech, China), separated by 12% SDS-PAGE, and transferred to PVDF membranes. After blocking with 5% nonfat milk, membranes were incubated overnight at 4 °C with anti-VP28 antibody (1:2000 dilution; Abcam, UK) and anti-GAPDH (1:2000; Abcam, UK). Following incubation with horseradish peroxidase-conjugated secondary antibody (1:5000; Abcam, UK), proteins were detected using ECL solution (Thermo Fisher Scientific, USA) and imaged with a chemiluminescence detection system.

WSSV copy number quantification

rMnLec7 or rMnLec9 (500 μL, 500 μg/mL) was incubated with 500 μL WSSV (10^6 copies/mL) at room temperature for 30 min with gentle rotation, using rGST (500 μg/mL) as the control. Prawns were injected with 100 μL of mixture. Hemocytes from three prawns per group were collected at 48 hpi for DNA extraction. WSSV copy numbers were quantified by TaqMan qPCR using WSSV-specific primers and probe (WSSV-qF: 5’-TTGGTTTCAGCCCGAGATT-3’, WSSV-qR: 5’-CCTTGGTCAGCC CCTTGA-3’, WSSV-probe: 5’-FAM-TGCTGCCGTCTCCAA-TAMRA-3’; Table S1). Reactions (25 μL) contained 12.5 μL Premix ExTaq (TaKaRa, Japan), 0.5 μL each primer (10 μM), 1 μL probe (10 μM), 1 μL DNA template, and 9.5 μL ddH_2O. Cycling conditions: 95 °C for 1 min; 40 cycles of 95 °C for 30 s, 52 °C for 30 s, 72 °C for 30 s. For the standard curve, a plasmid containing the WSSV target sequence (positions 225,368–225,424) was serially diluted (10^8 to 10^1 copies/μL). Copy numbers were calculated against this standard curve. Three independent assays were performed.

In vivo bacterial clearance assay

Overnight cultures of *V. parahaemolyticus* were harvested and resuspended in PBS (3×10^7 CFU/mL). rMnLec7 or rMnLec9 (500 μL, 500 μg/mL) was incubated with 500 μL bacterial suspension for 30 min with rotation. Controls included bacteria incubated with rGST (500 μg/mL) or PBS. Each prawn received 100 μL of mixture. Hemolymph (500 μL) from three prawns per group was collected at 2, 15, and 30 min post-injection, mixed with 500 μL anticoagulant buffer, serially diluted in PBS, and plated (50 μL) on LB agar. Bacterial colonies were enumerated after overnight incubation at 37 °C. Experiments included triplicate biological replicates.

Survival rate assay

rMnLec7 or rMnLec9 (1 mL, 500 μg/mL) was incubated with 1 mL WSSV (10^6 copies/mL) or *V. parahaemolyticus* (3×10^7 CFU/mL) for 30 min. Thirty prawns per group were injected with 100 μL mixture, using rGST-protein-pathogen mixtures as controls. Mortality was recorded daily for 6 days post-challenge. Survival curves were generated using GraphPad Prism, with statistical significance (*P*-values) determined by log-rank test. Three independent experiments were performed.

Ethics Statement

The sampling protocol was approved by the Ethics Committee of Experimental Animals at Nanjing University of Information Science and Technology and adhered to the Animal Care Guidelines issued by the Ministry of Science and Technology (China). No wild animals were used in this study.

SI Reference

1. Livak KJ, Schmittgen TD. Analysis of relative gene expression data using real-time quantitative PCR and the 2(-Delta Delta C(T)) Method. Methods. 2001;25(4):402-8.
